# Rational Engineering of Chorismate-Related Pathways in *Saccharomyces cerevisiae* for Improving Tyrosol Production

**DOI:** 10.3389/fbioe.2019.00152

**Published:** 2019-07-03

**Authors:** Wei Guo, Qiulan Huang, Hao Liu, Shaoli Hou, Suhao Niu, Yi Jiang, Xiaoming Bao, Yu Shen, Xu Fang

**Affiliations:** ^1^State Key Laboratory of Microbial Technology, Shandong University, Qingdao, China; ^2^Key Laboratory of Industrial Fermentation Microbiology, Tianjin University of Science and Technology, Ministry of Education, Tianjin, China; ^3^Shandong Henglu Biological Technology Co. Ltd, Jinan, China; ^4^State Key Laboratory of Biobased Material and Green Papermaking, School of Bioengineering, Qilu University of Technology, Jinan, China

**Keywords:** tyrosol, Ehrlich pathway, shikimate pathway, chorismate, *Saccharomyces cerevisiae*

## Abstract

Tyrosol is extensively used in the pharmaceutical industry as an important natural product from plants. In this study, an exogenous pathway involved in catalyzing tyrosine to tyrosol was introduced into *Saccharomyces cerevisiae*. Furthermore, The pyruvate decarboxylase gene *pdc1* was deleted to redirect the flux distribution at the pyruvate node, and a bifunctional NAD^+^-dependent fused chorismate mutase/prephenate dehydrogenase from *E. coli* (*Ec*TyrA) and its' tyrosine inhibition resistant mutant (*Ec*TyrA^M53I/A354V^) were heterologously expression in *S. cerevisiae* to tuning up the chorismate metabolism effectively directed the metabolic flux toward tyrosol production. Finally, the tyrosol yield of the engineered strain GFT-4 was improved to 126.74 ± 6.70 mg/g DCW at 48 h, increased 440 times compared with that of the control strain GFT-0 (0.28 ± 0.01 mg/g DCW). The new synergetic engineering strategy developed in this study can be further applied to increase the production of high value-added aromatic compounds derived from aromatic amino acid or shikimate in *S. cerevisiae*.

## Introduction

Tyrosol is a phenethyl alcohol derivative known to have antioxidant and anti-inflammatory effects (Kim et al., 2017). Tyrosol is widely found in many traditional fermented foods such as wines (Lafka et al., 2007) and virgin olive oil (Celano et al., 2018). Tyrosol is an important pharmaceutical intermediate and can be used as a precursor of salidroside (Ma et al., 2012), icariside D2 (Liao et al., 2018; Torrens-Spence et al., 2018), and hydroxytyrosol (Allouche and Sayadi, 2005; Li et al., 2018) in the pharmaceutical industry. These drugs have activities against cardiovascular disease (Granados-Principal et al., 2010; Hu et al., 2014), cancer (Hu et al., 2010; Liu et al., 2012), and viruses (Wang et al., 2009). There are three industrial methods used to produce tyrosol. The first method is extracting tyrosol from a variety of natural plants such as olive and *Rhodiola rosea*. The second method is chemical synthesis, such as producing tyrosol with the toxic phenylethyl alcohol and aromatic amine that is high-cost and not environmentally friendly (Henry et al., 1950; Yamada and Fujii, 1963). The third method is biosynthesizing tyrosol in microorganisms from sugars (Bai et al., 2014; Jiang et al., 2018). In recent years, the biosynthesis of tyrosol has received increasing attention attributed to it being low-cost, effective for production, and environmentally friendly. Thus, producing tyrosol via biosynthetic pathways in microorganisms is sustainable and has good prospects for development.

There are two main natural pathways to biosynthesize tyrosol: the Ehrlich pathway in microbes and the tyrosol synthesis pathway in plants (Jiang et al., 2018; Torrens-Spence et al., 2018). In the Ehrlich pathway of *Saccharomyces cerevisiae*, phenylpyruvate decarboxylase ARO10 catalyzes the conversion of 4-hydroxyphenylpyruvate (4HPP) to 4-hydroxyphenylacetaldehyde (4HPAA), followed by the reduction of 4HPAA to tyrosol by alcohol dehydrogenases (ADHs) (Sentheshanmuganathan and Elsden, 1958). In the plant pathway, tyrosine is converted into 4HPAA by aromatic aldehyde synthase (AAS) (Kaminaga et al., 2006; Torrens-Spence et al., 2012, 2013), followed by the reduction of 4HPAA to tyrosol catalyzed by ADHs.

Compared to plants, producing tyrosol by microorganisms is preferable, as microorganisms are fast-growing and utilize cheap and widely available carbon sources. Recently, scientists have attempted to biosynthesize tyrosol in *E. coli* (Satoh et al., 2012; Bai et al., 2014; Chung et al., 2017; Xue et al., 2017). However, compared to *E. coli, S. cerevisiae* is a robust, endotoxin-free microbial host strain with a long history of use in food and pharmaceutical fermentation, and has a Generally Recognized as Safe (GRAS) designation from the U.S. Food and Drug Administration (FDA). Furthermore, tyrosol is a natural substance occurring in *S. cerevisiae* fermentation, which can be biosynthesized via the Ehrlich pathway. In *S. cerevisiae*, phosphoenolpyruvate (PEP) derived from glycolysis and erythrose-4-phosphate (E4P) derived from the pentose phosphate pathway (PPP) are two substrates of shikimate pathway (Suástegui et al., 2016). Chorismate is the common metabolic precursor for the production of aromatic amino acids and their derivatives, and can be further converted into tyrosol via chorismate metabolism and Ehrlich pathway. It has been reported that overexpression of the prephenate dehydrogenase TYR1, the tyrosine feedback-resistant 3-deoxy-D-arabino-heptulosonate-7-phosphate (DAHP) synthase ARO4^K229L^, the tyrosine feedback-resistant chorismate mutase ARO7^G141S^ and shikimate kinase AROL involved in the catalytic reactions of shikimate improve the yields of tyrosine and tyrosol (Gold et al., 2015; Jiang et al., 2018).

Currently, there are only a few reports of biosynthesizing tyrosol in *S. cerevisiae* (Jiang et al., 2018; Torrens-Spence et al., 2018). It is necessary to develop more engineering strategies to further improve the production of tyrosol in *S. cerevisiae* and expand the biosynthesis of tyrosol from the lab-scale to the industrial scale.

In this study, we modified three modules on the pathway of tyrosol biosynthesis in *S. cerevisiae*. First, the Ehrlich Pathway was rewired by introducing the exogenous pathway for directly converting tyrosine to tyrosol; second, *pdc1* was disrupted to reduce the carbon flux below the pyruvate node; third, the upstream pathway of the chorismate metabolism was enhanced. Ultimately, a 440-fold increase in tyrosol yield was obtained compared to the strain harboring empty vector.

## Materials and Methods

### Strains and Culture Conditions

The *S. cerevisiae* laboratory strain BY4741 was used as a parent strain in this study. *E. coli* strain GB05-dir, a CcdB-sensitive strain, was used to construct the recombinant plasmids by Red-ET recombineering (Fu et al., 2012). The wild type *S. cerevisiae* strain BY4741 was incubated in YPD medium containing 10 g/L yeast extract, 20 g/L tryptone, and 20 g/L glucose. All strains engineered from the parent strain BY4741 were incubated in synthetic complete drop-out medium lacking uracil (SC-Ura) containing 1.77 g/L yeast nitrogen base without amino acids, 1.29 g/L DO supplement-Ura, and 20 g/L glucose as the sole carbon source. Yeast nitrogen base without amino acids was purchased from Solarbio (Beijing, China), DO supplement-Ura was purchased from Coolaber (Beijing, China), and other chemicals were purchased from Sinopharm Chemical Reagent Co, Ltd (Shanghai, China). All engineered *S. cerevisiae* strains were cultured in 300 mL shaking flasks containing 50 mL SC-Ura medium at 30°C with constant orbital shaking at 200 rpm.

### Construction and Transformation of Plasmids and Linearized Expression Cassettes

The primers used in this study were listed in Supplementary Table 3. All the promoters and terminators were amplified from *S. cerevisiae* BY4741 genomic DNA. The codon-optimized alcohol dehydrogenase (GenBank: WP_015958513.1) gene from *E. coli* (*EcAD*H^*syn*^) was synthesized and ligated with the *TEF1* promoter and *PGK1* terminator via fusion PCR, resulting in the fragment P_*TEF*1_*-EcAD*H^*syn*^*-*T_*PGK*1_. The codon-optimized AAS (GenBank: AAA33860.1) gene from *Petroselinum crispum* (*PcAA*S^*syn*^) was synthesized and ligated with the *TDH3* promoter and *CYC1* terminator via fusion PCR, resulting in the fragment P_*TDH*3_*-PcAA*S^*syn*^*-*T_*CYC*1_. The fragments P_*TDH*3_*-PcAA*S^*syn*^*-*T_*CYC*1_ and P_*TEF*1_*-EcAD*H^*syn*^*-* T_*PGK*1_ were inserted into plasmid pJFE3 by Red-ET recombineering, generating the recombinant plasmid pT1. The *pdc1* upstream and *pdc1* downstream fragments were amplified using the genomic DNA of *S. cerevisiae* BY4741 as a template. The linearized expression cassette pT2 was constructed by ligating the fragments *pdc1* upstream (500 bp), *kanMX*, and *pdc1* downstream (500 bp) via fusion PCR. The fragment *EcTyrA-*T_*CYC*1_was constructed by ligating chorismate mutase/prephenate dehydrogenase (Genbank: WP_103849370.1) gene sequence amplified from the *E. coli* genome and *CYC1* terminator via fusion PCR. The fragment *EcTyrA-*T_*CYC*1_ was inserted into pT2 under the *PDC1* promoter, resulting in the *EcTyrA* expression cassette pT3. The *EcTyr*A^*M*53*I*/*A*354*V*^ expression cassette pT4 was constructed based on pT3 using PCR-based site-directed mutagenesis. All the plasmids and linearized expression cassettes used or constructed in this study were listed in Supplementary Table 1. The plasmids and linearized expression cassettes were sequenced before transforming them into *S. cerevisiae* BY4741 via lithium acetate-mediated transformation (Schiestl and Gietz, 1989; Gietz et al., 1995). All of the strains used and constructed in this study were listed in Supplementary Table 2.

### Measurement of Cell Growth by Optical Density and Dry Cell Weight

The cell growth of *S. cerevisiae* was determined by measuring the OD_600_ value with a UV/VIS spectrophotometer (2802 UV/VIS Spectrophotometer, Unico Inc., USA). Cell concentration was calculated from a standard curve relating OD_600_ to dry cell weight (1 OD600 = 0.2959 g DCW/L).

### The Identification and Quantification of Tyrosol

The fermentation broth of *S. cerevisiae* was centrifuged at 10,732.8 × g for 2 min at 4°C, and then filtered with a 0.22 μm organic phase membrane. The standard tyrosol was purchased from Sigma-Aldrich (St. Louis, USA). Tyrosol concentration in the supernatant and standard tyrosol were analyzed and quantified by the Hitachi Elite LaChrom HPLC System (HITACHI, Japan) equipped with a C18 reverse-phase chromatography column (InertSustain C18 column, 250 × 4.6 mm, 5 μm) and Diode Array Detector. The HPLC conditions were according to a previous study (Satoh et al., 2012). The retention time of tyrosol was 19.05 min in the HPLC chromatogram. High-performance liquid chromatography-mass spectrometry (HPLC-MS) was performed on a Thermo Scientific Dionex UltiMate 3000 LC system and a Maxis Impact HD Mass Spectrometer with an ESI ionization probe. The positive mode was used to detect tyrosol in the mass range of 50–700 m/z.

## Results

### Design of Rational Metabolic Engineering to Increase the Tyrosol Production in *S. cerevisiae*

**Figure 1:**
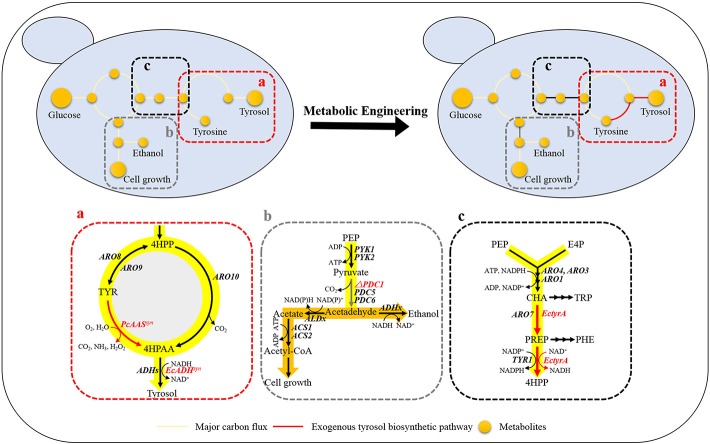
Design of rational metabolic engineering to enhance tyrosol production in *S. cerevisiae*. Metabolites: 4HPP, 4-hydroxyphenylpyruvate; TYR, tyrosine; 4HPAA, 4-hydroxyphenylacetaldehyde; PEP, phosphoenolpyruvate; Acetyl-CoA, acetyl coenzyme A; E4P, erythrose-4-phosphate; CHA, chorismate; PREP, prephenate; TRP, tryptophan; and PHE, phenylalanine. Genes: aminotransferase gene (*ARO8* and *ARO9*); phenylpyruvate decarboxylase gene(*ARO10*); pyruvate kinase gene (*PYK1* and *PYK2*); pyruvate decarboxylase genes (*PDC1, PDC*5, and *PDC*6); chorismate mutase gene (*ARO7*); 3-deoxy-7-phosphoheptulonate synthase (*ARO4* and *ARO3*); pentafunctional AROM polypeptide (*ARO1*); the NADP^+^-dependent prephenate dehydrogenase gene (*TYR1*); alcohol dehydrogenase genes (*ADHs*). The codon-optimized aromatic aldehyde synthase gene from *Petroselinum crispum* (*PcAA*S^*syn*^) and the NADH-dependent aryl-alcohol dehydrogenase gene from *E. coli* (*EcADH*^*syn*^) were heterologously expressed in *S. cerevisiae* to convert tyrosine to 4HPAA **(a)**, the native pyruvate decarboxylase gene *PDC1* was knocked out for tuning down the carbon flux below the pyruvate node **(b)**, the NAD^+^-dependent fused chorismate mutase/prephenate dehydrogenase gene from *Escherichia coli* (*EctyrA*) was expressed heterologously in *S. cerevisiae* to enhance choristmate metabolism **(c)**.

**Figure 2:**
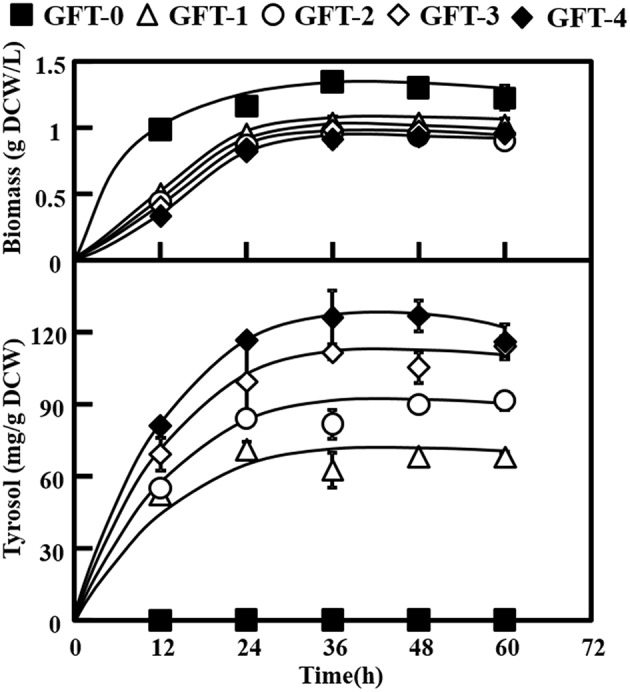
Effects of metabolic engineering of *Saccharomyces cerevisiae* on tyrosol and biomass. The engineered strains (GFT-1, GFT-2, GFT-3, and GFT-4) and control strain harboring the empty vector pJFE3 (GFT-0) were cultivated in SC-Ura medium with 2% glucose for 48 h. All experiments were performed using three biological replicates.

Tyrosol can be biosynthesized natively in *S. cerevisiae* via the endogenous pathway. The laboratory *S. cerevisiae* strain BY4741 was transformed with empty plasmid pJFE3, generating the control strain GFT-0. After 48 h of cultivation of GFT-0, 0.28 ± 0.01 mg/g DCW of tyrosol was detected in the medium (Figure 2). To improve the tyrosol yield of *S. cerevisiae*, we modified three modules to reprogram *S. cerevisiae* (Figure 1). First, the Ehrlich pathway was rewired (Figure 1a). Second, *pdc1* in *S. cerevisiae* was disrupted to redirect the flux distribution at the pyruvate node (Figure 1b). Finally, the upstream pathway of the chorismate metabolism was engineered (Figure 1c).

### Rewiring the Ehrlich Pathway in *S. cerevisiae*

The plasmid pT1 was transformed into *S. cerevisiae* BY4741 strain, resulting in strain GFT-1. Differing from the control strain GFT-0 with an empty plasmid pJFE3, tyrosine was converted into tyrosol directly catalyzed by PcAAS and the step from 4HPAA into tyrosol was strengthened in strain GFT-1 (Figure 1a).

As shown in Figure 2, strain GFT-1 produced 67.87 ± 0.60 mg/g DCW tyrosol after 48 h of shake flask cultivation, a 236-fold improvement compared to strain GFT-0. Thus, the introduction of the artificial *Pc*AAS^*syn*^-*Ec*ADH^*syn*^ pathway greatly increased the tyrosol yield of *S. cerevisiae*.

### Disrupting *pdc1* in *S. cerevisiae*

We aimed to disrupt the pyruvate decarboxylase gene *pdc1* to reduce the flux distribution at the pyruvate node and redirect the metabolic flux from PEP to the desired product tyrosol rather than toward ethanol and cell growth (Figure 1b). PDC1 was one of the main enzymes of the three pyruvate decarboxylases (PDC1, PDC5, and PDC6) (Wang et al., 2015). Therefore, *pdc1* in strain GFT-1 was disrupted, generating strain GFT-2. The tyrosol yield of strain GFT-2 reached 89.92 ± 1.48 mg/g DCW after 48 h of shake flask cultivation, and was significantly improved by 32.49%, compared to that of strain GFT-1 (Figure 2). Meanwhile, there was no obvious change between the biomass of strains GFT-1 and GFT-2 (Figure 2) and no changes was observed on ethanol production (Data not shown). These results proved that disruption of *pdc1* in *S. cerevisiae* increased the tyrosol production and has no significant influence on the ethanol fermentation and the cell growth.

### Engineering the Upstream Pathway of the Chorismate Metabolism in *S. cerevisiae*

**Figure 3:**
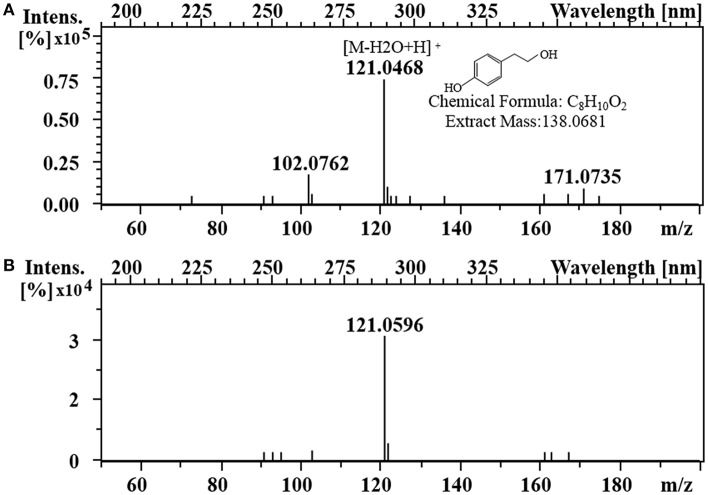
Identification of tyrosol. LC-MS analysis of standard tyrosol **(A)** and fermentation supernatant extracts **(B)**.

To enhance the upstream pathway of chorismate metabolism, a bifunctional chorismate mutase/ prephenate dehydrogenase gene from *E. coli* (*EcTyrA*) was heterologously expressed in *S. cerevisiae* (Figure 1c). The NAD^+^-dependent *Ec*TyrA catalyzes the rearrangement of chorismate to prephenate (PREP) as well as the oxidative decarboxylation of PREP to 4 HPP, and *Ec*TyrA^M53I/A354V^ was resistant to tyrosine inhibition (Lütke-Eversloh and Stephanopoulos, 2005; Lutke-Eversloh and Stephanopoulos, 2007). Therefore, the TyrA from *E. coli* (*EcTyrA*) or its' tyrosine-insensitive variant (*EcTyr*A^*M*53*I*/*A*354*V*^) was inserted into the *pdc1* site in strain GFT-1 to enhance chorismate metabolism, generating the strain GFT-3 and GFT-4. The tyrosol yield of the strain GFT-3 and GFT-4 reached 105.13 ± 6.28 mg/g DCW and 126.74 ± 6.70 mg/g DCW after 48 h of shake flask cultivation, and were improved by 16.91 and 40.94%, respectively, compared to that of strain GFT-2 (Figure 2). However, there was no significant change in biomass among strains GFT-2, GFT-3, and GFT-4 (Figure 2). These results suggested that heterologously expressing *EcTyrA* or *EcTyr*A^*M*53*I*/*A*354*V*^ in *S. cerevisiae* could enhance the upstream of chorismate metabolism and improve the tyrosol production. Finally, HPLC-MS was applied to identify the tyrosol in the fermentation broth, and the peak had a molecular ion at 121.0468 ([M-H2O+H]^+^) was corresponding to the standard tyrosol (m/z = 121.0596) (Figure 3).

## Discussion

*S. cerevisiae* is a commonly used industrial microorganism and a promising cell factory for the production of valuable natural products. Though *S. cerevisiae* produces tyrosol naturally with the Ehrlich pathway, the yield of tyrosol is rather low. In this study, we modified three modules involved in tyrosol biosynthesis to effectively improve the tyrosol production of *S. cerevisiae* (Figure 1).

In the Ehrlich pathway of *S. cerevisiae*, 4-hydroxyphenylpyruvate (4HPP) is an important intermediate, which is not only directly converted into 4HPAA but also is reversibly converted into tyrosine, and the flux distribution at the 4HPP node is not beneficial for the production of tyrosol. The competitive byproduct tyrosine from the 4HPP node could not be converted into tyrosol with the endogenous Ehrlich pathway in *S. cerevisiae*. Moreover, DAHP synthase ARO4 and chorismate mutase ARO7 play important roles in amino acid metabolism and both are strongly feedback inhibited by tyrosine accumulated in *S. cerevisiae* (Gold et al., 2015). In this study, the Ehrlich pathway in GFT-1 was rewired by introducing PcAAS that could convert tyrosine into 4HPAA, and then 4HPAA was synthesized via the artificial pathway and native tyrosol biosynthetic pathway simultaneously (Figure 1a). As shown in Figure 2, tyrosol yield of GFT-1 was greatly improved, compared to strain GFT-0 with empty pJFE3 vector. The result are consistent with those of others (Jiang et al., 2018; Torrens-Spence et al., 2018).

It was pointed out that competing fermentation pathways in engineered hosts should be eliminated to increase the conversion rate of feedstock to product (Aslan et al., 2017). PEP is a key central metabolism intermediate involved in glycolysis in *S. cerevisiae* and one of the precursors of shikimate pathway and pyruvate metabolism. The major carbon flux from the PEP node was directed to pyruvate and then used for cell growth and ethanol fermentation, whereas the minor proportion went to the shikimate pathway followed by the aromatic acids biosynthesis pathway (Gottardi et al., 2017). In *S. cerevisiae*, there are three main pyruvate decarboxylase genes (*pdc1, pdc5*, and *pdc6*). It was proved that the yeast cannot grow on medium with glucose as the single carbon source after disrupting all *pdc1, pdc5*, and *pdc6* (Flikweert et al., 1996). It was reported that disruption of *pdc1* caused a 30% decrease in pyruvate decarboxylase activity (Wang et al., 2015), and, on the contrary, enhanced the expression level of *pdc5* and respiratory metabolism (Eberhardt et al., 1999; Gottardi et al., 2017). In this study, *pdc1* in *S. cerevisiae* was disrupted and the tyrosol production of strain GFT-2 was significantly improved by 32.50% (Figure 2). We speculated that two reasons contribute to the improvement of tyrosol production in *S. cerevisiae*: (1) Disruption of *pdc1* led to a stronger respiratory metabolism that improved the ATP amount. Finally, the availability of PEP to shikimate pathway was increased owe to the increase of the ATP amount (Gottardi et al., 2017). (2) Disruption of *pdc1* resulted in the increase of expression level of *pdc5*, and PDC5 could compensate for the PDC activity of *S. cerevisiae pdc1*Δ strain (Wang et al., 2015). Differed from PDC1 and PDC6, PDC5 had the same catalytic ability as well as ARO10 that decarboxylated the aromatic substrate phenylpyruvate into phenylethanol (Romagnoli et al., 2012). Thus, we speculated that higher expression of *pdc5* might improve the conversion from 4HPP to 4HPAA. Our work suggested that disruption of *pdc1* in *S. cerevisiae* was a suitable strategy to improve tyrosol production.

Chorismate, which is a major branch point intermediate metabolite in biosynthesis pathways of aromatic acids and phenolic compounds, is synthesized by the shikimate pathway (Thompson et al., 2015). In *S. cerevisiae*, chorismate is consecutively converted by chorismate mutase ARO7 and prephenate dehydrogenase TYR1 to 4HPP via PREP (Rodriguez et al., 2015). The activity of ARO7 was allosterically inhibited by tyrosine and the conversion from PREP to 4HPP catalyzed by TYR1, which was reported as a bottleneck of the _L_-tyrosine synthetic pathway in *S. cerevisiae* (Mao et al., 2017). TyrA from *E. coli* (*Ec*TyrA*)* was a bifunctional NAD^+^-dependent fused chorismate mutase/prephenate dehydrogenase (Bhosale et al., 1982) and overexpression of *TyrA* improved tyrosine production in *E. coli* (Lutke-Eversloh and Stephanopoulos, 2007). Furthermore, *Ec*TyrA was reported to be a homodimer consisted of CM domain and PDH domain, and was regulated by the feedback inhibition of tyrosine. The variant *Ec*TyrA^M53I/A354V^ was resistant to the inhibition of tyrosine (Lütke-Eversloh and Stephanopoulos, 2005). In this study, the genes *EcTyrA* or its' variant—*EcTyr*A^*M*53*I*/*A*354*V*^ was inserted into the *pdc1* site in strain GFT-2 and tyrosol production of strain GFT-3 and GFT-4 were improved by 16.91 and 40.94%, respectively, compared to that of strain GFT-2. Moreover, the improvement of tyrosol yield with heterologously expressing *EcTyr*A^*M*53*I*/*A*354*V*^ in *S. cerevisiae* was higher than that of *EcTyrA* (Figure 2). We hypothesized that chorismate metabolism followed by the Ehrlich pathway might be strengthened and the inhibition from tyrosine was alleviated by expressing *EcTyr*A^*M*53*I*/*A*354*V*^ in *S. cerevisiae*.

Differing from previous work by Jiang et al. (2018), our study showed that the tyrosol yield was significantly improved up to 86.74% owe to the introduction of *EcTyr*A^*M*53*I*/*A*354*V*^ and the disruption of *pdc1* simultaneously based on the expression of *PcAA*S^*syn*^ (Figure 2).

In this study, it was proved that the engineering *S. cerevisiae* with the modification of three modules was more suitable for tyrosol production. The new engineering strategy could also be further applied to enhance the production of aromatic amino acids and their derivatives in *S. cerevisiae*.

## Data Availability

The raw data supporting the conclusions of this manuscript will be made available by the authors, without undue reservation, to any qualified researcher.

## Author Contributions

WG, QH, SH, and SN performed experiments. WG and XF wrote the manuscript and conceived the study. WG, HL, YJ, XB, YS, and XF were involved in analysis and interpretation of experimental data. XF and HL coordinated the project.

### Conflict of Interest Statement

SH was employed by company Shandong Henglu Biological Technology Co. Ltd. The remaining authors declare that the research was conducted in the absence of any commercial or financial relationships that could be construed as a potential conflict of interest.
