# Supplementary material for: Rational Engineering of Chorismate-Related Pathways in Saccharomyces cerevisiae for Improving Tyrosol Production
Source: Front Bioeng Biotechnol. 2019 Jul 3;7:152. doi: 10.3389/fbioe.2019.00152 (PMC6616077; doi:10.3389/fbioe.2019.00152)
Supplement: Supplementary file 1 [file Data_Sheet_1.PDF]

Supplementary Material

Supplementary Table 1 Plasmids and cassettes used in this study

Plasmids/cassettes	Features	Source/Reference
pJFE3	URA3; 2μm ori with P_{TEF1}, T_{PGK1}; *Amp*	Shen et al. (2012)
pT1	pJFE3 carrying P_{TDH3}-*PcAAS*^{syn}-T_{CYC1} -P_{TEF1}-*EcADH*^{syn}-T_{PGK1} cassette	This study
pT2	*pdc1* upstream-*kanMX*-*pdc1* downstream	This study
pT3	*pdc1* upstream-*EcTyrA*-T_{CYC1}-*kanMX*-*pdc1* downstream	This study
pT4	*pdc1* upstream-*EcTyrA*$^{M53I/A354V}$ -T_{CYC1}-*kanMX*-*pdc1* downstream	This study

Supplementary Table 2 Strains used in this study

Strains	Descriptions	Source/Reference
BY4741	*MATa*, *his3⊿1*, *leu2⊿0*, *met5⊿0* and *ura3⊿0*	EUROSCARF
GFT-0	BY4741 carrying plasmid pJFE3	This study
GFT-1	BY4741 carrying plasmid pT1	This study
GFT-2	GFT-1 with *pdc1* disrupted	This study
GFT-3	GFT-1 with *pdc1* disrupted and *EcTyrA* expressed	This study
GFT-4	GFT-1 with *pdc1* disrupted and $EcTyrA^{M53I/A354V}$ expressed	This study

EUROSCARF, European *Saccharomyces cerevisiae* Archives for Functional Analysis.

Supplementary Table 3 Oligonucleotides primers used in construction of plasmids and cassettes

Primer	Oligonucleotides (5’ → 3’)
4CYCT-F	AAATTCTGCGTTCGTTAAAGCTTGCAAATTAAAGCCTTCG AGCGTCC
4CYCT-R	CAAGTTGGTCGAGGTCTTGTCTTAATCATGTAATTAGTTAT GTCACGCTT
4PcAAS-F	AAGCGTGACATAACTAATTACATGATTAAGACAAGACCTC GACCAACTTG
4PcAAS-R	GAATAAACACACATAAACAAACAAAATGGGTTCCATTGA TAACTTGACCG
4TDH3-F	CGGTCAAGTTATCAATGGAACCCATTTTGTTTGTTTATGTG TGTTTATTC
2PTDH3-R	AAACAGCTATGACCATGATTACGCCTCATTATCAATACTG CCATTTCAAA
2V-F	TTTGAAATGGCAGTATTGATAATGAGGCGTAATCATGGTC ATAGCTGTTT
3V-R	CTCTTATTGACCACACCTCTACCGGGGCGTAATCATGGTC ATAGCTGTTT
PDC1U-F	TTATGTATGCTCTTCTGACTTTTCG
PDC1U-R	GGGACGAGGCAAGCTAAACAGATCTTTTGATTGATTTGAC TGTGTTATTT
G418-F	AAATAACACAGTCAAATCAATCAAAAGATCTGTTTAGCTT GCCTCGTCCC

G418-R	TATAAAACTTTAACTAATAATTAGAGAGAGCTCGTTTTCG ACACTGGATG
PDC1D-F	CATCCAGTGTCGAAAACGAGCTCTCTCTAATTATTAGTTA AAGTTTTATA
PDC1D-R	TTTCAATCATTGGAGCAATCATT
2PDC1U-F	TTATGTATGCTCTTCTGACTTTTCG
2PDC1U-R	AATGCGGTCAATTCAGCAACCATTTTGATTGATTTGACTG TGTTATT
tyrA1-F	AATAACACAGTCAAATCAATCAAAATGGTTGCTGAATTGA CCGCATT
tyrA1-R	ACATAACTAATTACATGATTAATTAATTACTGGCGATTGT CATTCGC
4TCYC1-F	GCGAATGACAATCGCCAGTAATTAATTAATCATGTAATTA GTTATGT
4TCYC1-R	GGGACGAGGCAAGCTAAACAGATCTGGCGCGCAAATTAA AGCCTTCGAGC
2G418-F	GCTCGAAGGCTTTAATTTGCGCGCCAGATCTGTTTAGCTT GCCTCGTCCC
2G418-R	AAAACTTTAACTAATAATTAGAGAGAGCTCGTTTTCGACA CTGGATGG
2PDC1D-F	CCATCCAGTGTCGAAAACGAGCTCTCTCTAATTATTAGTT AAAGTTTT
2PDC1D-R	TTTCAATCATTGGAGCAATCATTTT

TyrA-M53I-R	TTCCGCCTCTGCACGACGCGAGGCCAAAATAGATGCCTCG CGCTC
TyrA-M53I-F	CTATTTATGTTCCGGAGCGCGAGGCATCTATTTTGGC
TyrA-A354V-R	AATAACACGCGGCTTTCACTCTGAAAACGCTGAACGTAAT CGCCGAAC
TyrA-A354V-F	GTTCGGCGATTACGTTCAGCGTTTTCAGAGTGAAAGCCGC GTGTTATT
